# A Rare Case of Bullous Morphea Associated with Autoimmune Hepatitis

**DOI:** 10.3390/jcm13154356

**Published:** 2024-07-25

**Authors:** Jorinta Jokubaitė, Goda Klapatauskaitė, Monika Marta Macejevska, Jūratė Grigaitienė

**Affiliations:** 1 Clinic of Infectious Diseases and Dermatovenereology, Faculty of Medicine, Vilnius University, 03101 Vilnius, Lithuania; klapatauskaite.goda@gmail.com (G.K.); jurate.grigaitiene@gmail.com (J.G.); 2 Kardiolitos Klinikos, 05263 Vilnius, Lithuania; monika.macejevska@gmail.com

**Keywords:** bullous morphea, scleroderma, autoimmune hepatitis

## Abstract

(1) Background: Bullous morphea is an extremely rare form of localized scleroderma, a condition that is marked by the presence of sporadic and intermittent blisters on sclerodermatous skin. This condition stands out due to its rarity and the unique manifestation of blistering, which sets it apart from other forms of localized scleroderma. Due to the infrequent presentation of bullous morphea, there is a significant gap in our understanding of its pathogenesis. The exact mechanisms that lead to the development of this condition remain largely unknown, which poses a challenge for medical professionals in terms of both diagnosis and treatment. The limited number of reported cases makes it difficult to establish a standardized approach to managing this condition, and as a result, treatment options are often limited and may vary from one patient to another. (2) Methods: In this case report, we present a rare case of bullous morphea that manifested before the onset of autoimmune hepatitis. When morphea presents unusually or is resistant to traditional immunosuppressive treatment, a comprehensive assessment of possible concurrent autoimmune illnesses provoking the rash must be conducted. (3) Results: We report a successful case of bullous morphea treated with systemic corticosteroids following a diagnosis of autoimmune hepatitis. (4) Conclusions: This case highlights the importance of considering overlapping autoimmune conditions in the management of bullous morphea and the potential efficacy of systemic corticosteroids in such scenarios. Collaborative efforts involving dermatologists, rheumatologists, and hepatologists are essential to enhance understanding and optimize treatment outcomes for patients affected by this rare and complex condition. Thus, further research is necessary to gain a deeper understanding of the pathogenesis of bullous morphea and to develop more effective and targeted treatment options for patients affected by this condition.

## 1. Introduction

Morphea is a fibrosing condition of the skin, often known as localized scleroderma [1,2]. Bullous morphea impacts around 2.7 individuals per 100,000 annually, showing a higher incidence in females. While it can affect people of any ethnicity, those of white descent are particularly susceptible [3]. Typically, the condition manifests in the fifth decade of life, with the plaque form being the most prevalent and well researched. Individuals with a family history of autoimmune diseases are at a heightened risk of developing morphea. A recent history of insect bites, trauma, vaccinations, or surgeries may be relevant. Additionally, morphea has been reported to occur in association with several blistering disorders, such as pemphigus vulgaris, bullous pemphigoid, and porphyria cutanea tarda. A very uncommon form of localized scleroderma called bullous morphea is characterized by sporadic, intermittent blisters [1]. Bullous morphea is considered to be a multifactorial condition, with the most acknowledged explanation favoring lymphangiectasia secondary to lymphatic flow obstruction caused by dermal sclerosis and subsequent subepidermal edema [2,3]. However, the precise etiology behind the formation of bullae in bullous morphea remains incompletely understood. Notably, not all documented cases of bullous morphea have shown associated lymphangiectases. In this case report, we present a new case of bullous morphea that manifested before autoimmune hepatitis (AIH) and share our findings of dermal sclerosis, thereby contributing to the current knowledge of this rare entity. To our knowledge, this is the first documented case of bullous morphea associated with autoimmune hepatitis. By documenting this case, we aim to expand the current understanding and contribute to the literature on bullous morphea, emphasizing the importance of comprehensive diagnostic approaches in managing such complex dermatological conditions.

## 2. Case Presentation

A 60-year-old woman presented with a severe rash on her torso and progressive thickening of the skin over the past year. Despite undergoing prior therapy with 15 mg of methotrexate once a week for several months, the lesions had extended to involve her upper thighs and back.

**Figure 1:**
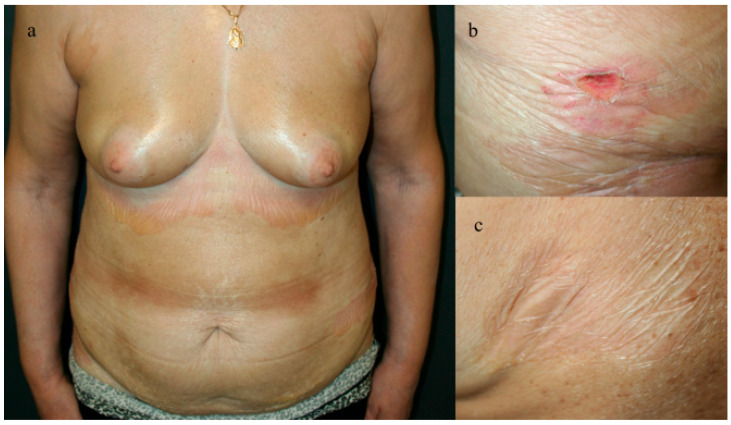
(**a**) The tense, shiny skin of the torso with widespread bullae in the inframammary region and lateral torso. (**b**) Close view of the eroded (**c**) and ivory-colored plaque.

During physical examination, the patient exhibited erythematous sclerodermatous plaques adorned with tense bullae in areas including the lower abdomen, inframammary region, lateral torso, and upper thighs. The lateral torso displayed scaly, painful plaques with central erosions, alongside brownish sclerotic plaques centrally located on the torso. Additionally, atrophic, well-defined, ivory-colored plaques were observed on the back and calves. On palpation, the patient’s skin was notably tight and shiny (Figure 1). The dermoscopy of the plaques showed characteristic morphea features such shiny fibrosis patches and linear blood vessels, whereas the dermoscopy of the bullous lesions showed erythema surrounding the bullous skin lesions and serous fluid within the lesions. This clinical presentation suggested a complex manifestation of bullous morphea, characterized by a combination of sclerodermatous plaques, bullae formation, erosions, and atrophic changes across various body regions. The extensive involvement despite previous treatment attempts highlighted the challenging nature of managing this rare and debilitating condition.

**Figure 2:**
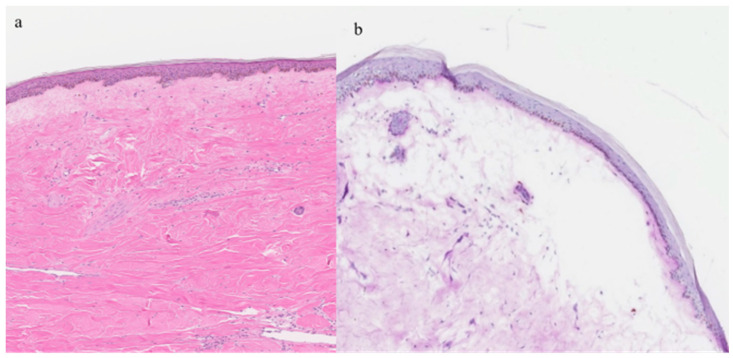
(**a**) Epidermal atrophy and thickened homogenous collagen bundles in the dermis, with mild and diffuse mononuclear cell infiltration (4X-HE). (**b**) Subepidermal detachment in a localized region, accompanied by papillary dermal edema (10X-PAS).

The patient was hospitalized for further examination and underwent extensive laboratory investigations, which included a broad spectrum of general laboratory tests such as complete blood count, comprehensive metabolic panel, liver function tests, renal function tests and thyroid panel. Additionally, autoimmune panels and Borrelia serology were performed to evaluate specific autoimmune markers and screen for Lyme disease, respectively. Further assessments included ultrasound examinations of internal organs and a chest X-ray to exclude any paraneoplastic processes and provide an overall assessment of the patient’s health status. None of the aforementioned tests yielded remarkable results. Notably, the blood serum antinuclear antibody test returned positive for anti-DFS70 antibodies, indicating a specific autoimmune marker. Other laboratory tests did not reveal any significant abnormalities. Two punch biopsies, each with a diameter of 4 mm, were obtained from a bullous lower thoracic plaque. Histopathological examination revealed alterations consistent with morphea (Figure 2). The histological examination revealed epidermal thinning, thickened and uniform collagen bundles in the dermis, and mild diffuse infiltration of mononuclear cells. Additionally, there was observed subepidermal detachment in a specific area and papillary dermal edema. No lymphangiectasia was noted. The results of direct immunofluorescence were negative. Given the aforementioned results, the patient was diagnosed with bullous morphea.

Systemic scleroderma was definitively excluded following thorough assessment through laboratory tests, clinical examination, and consultation with a rheumatologist. Initially, the patient received an initial dose of 8 mg of intravenous dexamethasone, which was subsequently tapered off. This was followed by a regimen of 25 mg of oral prednisone, gradually tapered to 5 mg daily over a span of 5 weeks. Despite these efforts, the skin condition continued to deteriorate. In response, systemic PUVA therapy was introduced during the same hospital stay, involving 20 mg of methoxsalen and betamethasone ointment applied daily. However, this treatment yielded unsatisfactory results. Subsequently, a combination therapy of 10 mg of oral prednisone and 1000 mg of mycophenolate mofetil daily was attempted, but also proved unsuccessful. Over the following year, the patient underwent regular follow-up visits with a dermatovenerologist every three months. Throughout this period, continuous treatment with low-dose corticosteroids was maintained in an effort to manage and alleviate the persistent symptoms of the condition.

**Figure 3:**
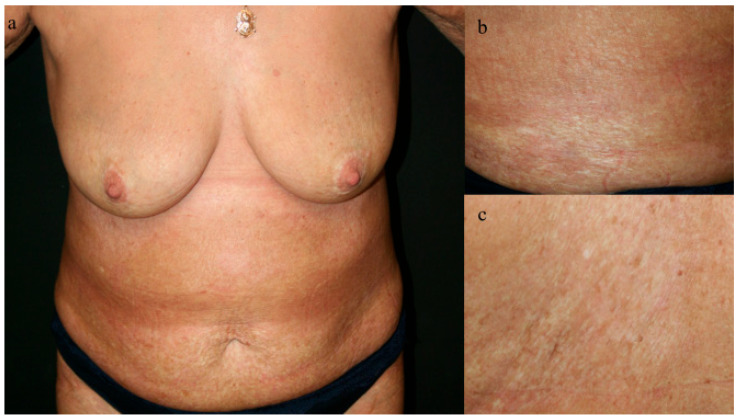
(**a**–**c**) The treatment outcomes after one and a half years in the specific skin areas shown in Figure 1.

During one of the dermatology outpatient visits, the patient reported experiencing a moderate episode of jaundice. Subsequently, the patient was referred to a gastroenterologist for further evaluation. Tests for viral hepatitis yielded negative results, but liver transaminase levels were notably elevated (AST: 731 U/L, ALT: 903 U/L). A subsequent liver biopsy confirmed the diagnosis of autoimmune hepatitis (AIH). In response to the diagnosis, the treatment plan was initiated, which involved starting the patient on 9 mg of systemic budesonide with a gradual tapering of the dose over time. Over the course of three months, this therapeutic approach resulted in significant improvements in both hepatic function and the patient’s skin condition. Positive outcomes observed included a reduction in skin tension, healing of erosions, and the absence of bullae on the plaques (Figure 3). To date, the patient has remained in remission for both conditions with the use of low-dose systemic steroids, according to the latest information available. This case underscores the effective management of AIH with budesonide and highlights the correlation between hepatic and dermatologic manifestations in autoimmune diseases.

## 3. Discussion

According to the literature, fewer than 100 cases of bullous morphea have been reported up to this day [2]. Morrow first reported a case of bullous morphea in 1896 [4]. Since then, numerous theories on its origin have been proposed. Bullous morphea is considered to be a multifactorial condition, with the most acknowledged explanation favoring lymphangiectasia secondary to lymphatic flow obstruction caused by dermal sclerosis and subsequent subepidermal edema. However, none of these theories provide a completely satisfactory explanation, as evidenced by the fact that some lesions of bullous morphea, such as in our case, do not exhibit lymphangiectasia upon histological examination, and not all cases of scleroderma with lymphangiectasia lead to blister formation. Local friction, trauma, radiation therapy, and immune-mediated inflammation play a significant role, particularly as bullous morphea tends to localize in intertriginous areas, where blisters can develop as an isomorphic skin response [2]. Our patient did not report any history of trauma; however, the lesions were located in areas prone to local friction, such as the bra and underwear regions, as well as on the legs where minor unnoticed trauma can occur.

The most common differential diagnosis in a such clinical scenario is bullous lichen sclerosus et atrophicus [5]. Despite some authors suggesting that these two conditions may exist on a spectrum, distinctions can be drawn based on their pathological characteristics. Morphea is characterized by fibrosis affecting deeper skin layers such as the reticular dermis and subdermal tissues, leading to thickening and hardening of the affected areas. In contrast, lichen sclerosus primarily targets the superficial papillary dermis and the epidermis, resulting in thinning and atrophy of the skin. The localization of these conditions also varies significantly, where morphea tends to favor the trunk, while lichen sclerosus et atrophicus predominantly affects the anogenital region [6]. However, there are documented cases where these conditions overlap, presenting challenges in clinical differentiation [5]. Also, morphea must be differentiated from other fibrotic skin conditions, such as lipodermatosclerosis, scleromyxedema, and post-irradiation morphea [7]. In the case described, histopathological findings revealed characteristics consistent with bullous morphea, emphasizing extensive involvement of the full thickness of the dermis, characterized by thickened, hyalinized collagen, as well as the formation of subepidermal bullae. Upon clinical evaluation, the lesions displayed a distinctive and characteristic pattern of distribution for bullous morphea.

Currently, there are no established guidelines for diagnosis and treatment for this variant of morphea due to its rarity, and the primary approach to treatment often involves immunosuppressive therapy. The standard evaluation in morphea patient group consists of complete blood count, inflammatory markers, anti-nuclear antibody, thyroid function tests, rheumatoid factor, or anti-CCP antibodies testing. In cases of bulla presence in morphea lesions, testing for blood markers of autoimmune bullous skin diseases should be performed if available. In regions where Lyme disease is endemic, such as Lithuania, testing for Borrelia serology should be a standard part of the diagnostic evaluation for patients presenting with morpheoform lesions. This approach ensures accurate diagnosis, appropriate treatment, and better overall patient management, considering the potential association between Borrelia spp. infection and morphea. Skin biopsy is preferred, at least two biopsies should be taken as autoimmune bullous diseases ought to be denied using immunofluorescence. Systemic sclerosis must be suspected in all patients until proven otherwise. Patients should be actively questioned for systemic symptoms such as joint and muscle pain, reflux, fatigue, cold hands and changes in the skin color of the fingers (suggestive of possible Raynaud’s phenomenon), and ocular symptoms of dryness or blurred vision.

Various options, such as local and systemic steroids, methotrexate, mycophenolate mofetil, colchicine, antimalarial drugs (f.e. hydroxychloroquine), and phototherapy (particularly broad band ultraviolet A, ultraviolet A1, and photochemotherapy) have been attempted in previously described cases as a treatment option [4,5]. If a Borrelia spp. infection is suspected in a patient with morphea, the appropriate antibiotic therapy for Lyme disease should be considered. Treatment of the underlying infection could potentially impact the course of morphea, although the effects of antibiotic therapy on morphea are still under investigation. Also, in our specific case, numerous treatment modalities were explored, but noteworthy improvement in the skin condition was observed only after the administration of potent systemic steroids for concomitant AIH.

With some exceptions, localized morphea seldom leads to severe systemic consequences, but there are occasional associations with autoimmune diseases such as rheumatoid arthritis, alopecia areata, and AIH. AIH is a chronic inflammatory liver disease characterized by immune-mediated destruction of hepatocytes. It is considered rare, affecting approximately 1 to 2 per 100,000 individuals annually, with a higher prevalence in women compared to men. AIH can occur at any age but commonly manifests between the ages of 15 and 40. There is variation in the clinical presentation of AIH. Acute icteric hepatitis affects about one-third of patients when they present for clinical care; fulminant hepatic failure may also occur on occasion. A majority of the patients have milder conditions, and some might have subclinical illnesses. Non-specific symptoms may bring them to the attention of a clinician. The most significant AIH-associated symptom is fatigue, which limits engagement in previously pleasurable or satisfying activities. Other symptoms include depression and anxiety, which are related to emotions of uncertainty, pessimism, and hopelessness, as well as sleep disturbances, neurological symptoms, and pain [8]. Elevated blood aminotransferases, increased levels of immunoglobulin G, the presence of specific autoantibodies, and the identification of interface hepatitis with plasma cell infiltration in liver biopsies are indicative of AIH [9]. The treatment aims to suppress immune activity and prevent progressive liver damage. Corticosteroids, such as prednisone, in combination with azathioprine are considered first-line therapy and effectively induce remission in many patients. However, a variety of side effects of steroid therapy, including weight gain, diabetes mellitus, hypertension, emotional instability, and even psychosis, necessitate lowering dosages or quitting the medication completely. After two years of treatment, 80% of patients experience steroid-related adverse effects; however, this is less common in combined therapy with azathioprine. To prevent osteoporosis from developing, patients should have their bone density measured before starting steroid therapy. Patients ought to also consider vitamin D supplements and get enough calcium in their diet according on their sex and age [10]. We present this case to emphasize the importance of conducting a comprehensive examination for concomitant autoimmune diseases in instances of atypical or treatment-resistant morphea.

## 4. Conclusions

In cases of atypical or treatment-resistant morphea, conducting a comprehensive assessment for associated autoimmune diseases is essential. This thorough evaluation is crucial for successful disease management, as identifying and addressing concurrent autoimmune conditions can significantly impact the treatment approach and outcomes. Recognizing these associations allows healthcare providers to tailor therapeutic strategies more effectively, potentially improving patient prognosis and quality of life. Therefore, a detailed investigation into possible autoimmune comorbidities should be an integral part of the diagnostic and treatment process for patients presenting with unusual or refractory morphea.

## Data Availability

The clinical records analyzed during this case report are not publicly available due to a duty of confidentiality but are available from the corresponding author on reasonable request. Our clinical record was collected in the clinical information system of the Vilnius University Hospital Santaros Klinikos.
